# Effect of role overload on missed nursing care in China: The role of work addiction and leader‐member exchange

**DOI:** 10.1002/nop2.1565

**Published:** 2022-12-23

**Authors:** Lin‐Li Xie, Wenchun Jiang, Eksiri Niyomsilp, Jie Jing, Lu Feng, Yilin Wen, Li Wang, Rongmei Zheng

**Affiliations:** ^1^ University of Electronic Science and Technology of China, Sichuan Provincial People's Hospital Chengdu China; ^2^ School of Medicine, University of Electronic Science and Technology of China Chengdu China; ^3^ Department of Nursing University of Electronic Science and Technology of China, Sichuan Provincial People's Hospital Chengdu China; ^4^ School of Management Shinawatra University Pathumthani Thailand; ^5^ Shehong Municipal Hospital of Traditional Chinese Medicine Shehong China; ^6^ The Second People's Hospital of Panzhihua Panzhihua China; ^7^ Guangyuan Third People's Hospital Guangyuan China

**Keywords:** leader‐member exchange, missed nursing care, nursing management, role overload, work addiction

## Abstract

**Aim:**

The aim was to examine the effect of role overload, work addiction and leader‐member exchange on missed nursing care.

**Design:**

A cross‐sectional study.

**Methods:**

Chinese registered nurses from five Sichuan province public hospitals were studied from March 2022–May 2022. The measurements were derived from a questionnaire on role overload, work addiction, missed nursing care, leader‐member exchange and a sociodemographic datasheet. Descriptive statistics and inferential statistics were conducted (*N* = 403).

**Results:**

Role overload was associated with missed nursing care, and work addiction played a mediation role. Leader‐member exchange negatively predicted work addiction and played a moderating role between role overload and work addiction. The effect of role overload on work addiction was attenuated when the level of leader‐member exchange was higher. Promoting leader‐member exchange could mitigate how role overload undermines missed nursing care.

## 1 BACKGROUND

In recent years, missed nursing care has aroused global concern (Willis et al., 2021). Missed nursing care is the omission or noticeable delay of care measures patients need for various reasons (Kalisch et al., 2009). It significantly affects patient safety and quality of care and leads to adverse events (Andersson et al., 2022; Nymark et al., 2022). Missed nursing care is linked to a lack of human resources, physical resources, and communication (Zhao et al., 2020). One of the essential work‐related antecedents is the nursing work environment (Dutra & Guirardello, 2021; Smith et al., 2018).

Work stress is common in the nursing work environment. Role stress as an important branch of it has attracted increasing attention from nursing scholars. Established studies have shown that role stress mainly arises from role conflict, role ambiguity and role overload (Kahn et al., 1964; Peterson et al., 1995). Role overload is one of the severe challenges in the nursing work environment (Wang & Li, 2021). Role overload refers to the role stress perceived by individuals when they lack the necessary competencies, abilities or sufficient time to fulfil diversity role requirements (Rizzo et al., 1970). It means that individuals take on a role that demands an excessive amount of effort (Schaubroeck et al., 1989; Stevenson & Duxbury, 2019). When the actual demands on the “nurse” role are beyond the nurse's expectation, the role overload of nurses arises. With the spread of COVID‐19 across the globe, the shortage of nurses is becoming increasingly apparent. Role overload is a common problem among nurses in China (Zhang et al., 2021). Role overload is proven to negatively affect employees and their organizations, including a reduction in engagement and a deterioration in missed nursing care conditions (Zhang et al., 2022). The research exploring how to reduce nursing missed care in a high‐pressure nursing work environment has gradually become a hot topic of nursing management research in recent years. However, no consensus has been reached on the impact of role overload on missed nursing care so far (Tang & Vandenberghe, 2021). And there is a limited exploration of how contextual factors reduce missed nursing care through individual psychological mechanisms. Therefore, this study focused on the mechanisms of how nurse role overload impacts missed nursing care through work addiction and its boundary conditions.

The Job Demands‐Resource model (JD‐R) (Bakker & Demerouti, 2007) is one of the most influential models of workplace stress available (Berthelsen et al., 2018). The JD‐R suggests that through a pathway, job demands add to job stress and have an impact on organizational outcomes. Simultaneously, JD‐R also showed that job resources can mitigate the impact of job demands on stress and ultimately improve organizational outcomes (Nicholson, 2021).

According to the JD‐R (Bakker & Demerouti, 2007), the characteristics of employees' work can be divided into job demands and job resources. Job resources are the psychological, social and organizational resources associated with work. It helps individuals to save the physical or psychological costs of job requirements and stimulates individual growth and development to achieve work goals. However, job demands require employees to make efforts to achieve job goals, which will lead to the continuous depletion of individual resources (Chevalier et al., 2021). Based on the JD‐R model, role overload shows obstructive job demands (Crawford et al., 2010). Studies have confirmed that role overload hurt individual psychology (Srulovici & Yanovich, 2022).

Work addiction refers to an individual's work‐obsessed state in which an individual puts in so much effort far beyond what is required and expected at work that he or she neglects life outside work (Schaufeli et al., 2008). Most scholars consider work addiction to be a subjective out‐of‐control that includes a behavioural component of “overwork” and a cognitive component of compulsion to work (Andreassen, 2014). One of the critical antecedent variables of work addiction is stress (Aziz & Zickar, 2006). It has been found that the more stress nurses perceive, the higher their level of work addiction (Borges et al., 2021). Role overload is just one of the role stressors perceived by nurses; thus, we believe that role overload may be closely associated with work addiction.

Based on the JD‐R model, when dealing with obstructive job demands, at the cognitive level, employees feel they are unable to provide sufficient resources to achieve their work goals. At the behavioural level, role overload makes employees experience anxiety, generate negative moods and force them to put a lot of energy and effort into their work, which in turn leads to work addiction (Molino et al., 2016; Van Wijhe et al., 2011). The reason mentioned above makes the following hypotheses:

Role overload is positively correlated with missed nursing care.

Role overload is positively correlated with work addiction.

Regarding mechanisms, we suggest that work addiction is one underlying way in which role overload disrupts missed nursing care. The conservation of resources (COR) theory (Hobfoll, 1989) assumes that individuals seek to retain, conserve and create resources and that the actual or expected resource loss causes stress responses. When resources are limited, employees tend to protect existing resources from loss. When an employee feels that he or she has done the best, but the demands are still challenging to achieve, the employee tends to adjust the performance goal to a lower level so that no additional effort is required. Thus, continuous role overload will generate negative emotions and perceptions to cause passive and emotion‐centred negative work responses. These poor job responses could result in intensified missed nursing care (Clark et al., 2020). Consequently, we make the below hypothesis.

Work addiction is positively correlated with missed nursing care.

Work addiction plays a mediating role between role overload and missed nursing care.

The role of contextual factors in role overload effects has often been overlooked, even though it is essential in the research of missed nursing care. Contextual factors have been shown to cushion the negative relationship between role overload and affective commitment (Fisher, 2014). The JD‐R model indicates that there are job resources (e.g. leadership styles) that can reduce the path of energy consumption (Srulovici & Yanovich, 2022).

Leader‐member exchange relationship is considered a type of job resource in current studies (Tang & Vandenberghe, 2021), and it can bring benefits to individuals and organizations (Irshad et al., 2022). Leader‐member exchange is a kind of relationship exchange based on the social situation, and it is a social exchange between leaders and their subordinates in emotion, interests, cognition and behaviour (Graen & Uhl‐Bien, 1995). High‐quality leader‐member exchanges as contextual resources may prevent the resource‐draining processes associated with stressors and reduce the negative impact of job stressors on the quality of care. One research (Tang & Vandenberghe, 2021) showed that leader‐member exchange could play a buffer role in job performance decline caused by role overload. Thus, good leadership member exchange may be an important pathway to counteract the process of nurse resource drain, thereby allowing nurses to devote effective resources to caregiving and reduce missed nursing care. Based on the above arguments and evidence, this study proposes the following hypotheses:

Leader‐member exchange plays a moderating role between role overload and work addiction. Specifically, the effect of role overload on work addiction is weaker when the level of leader‐member exchange is higher.

**FIGURE 1:**
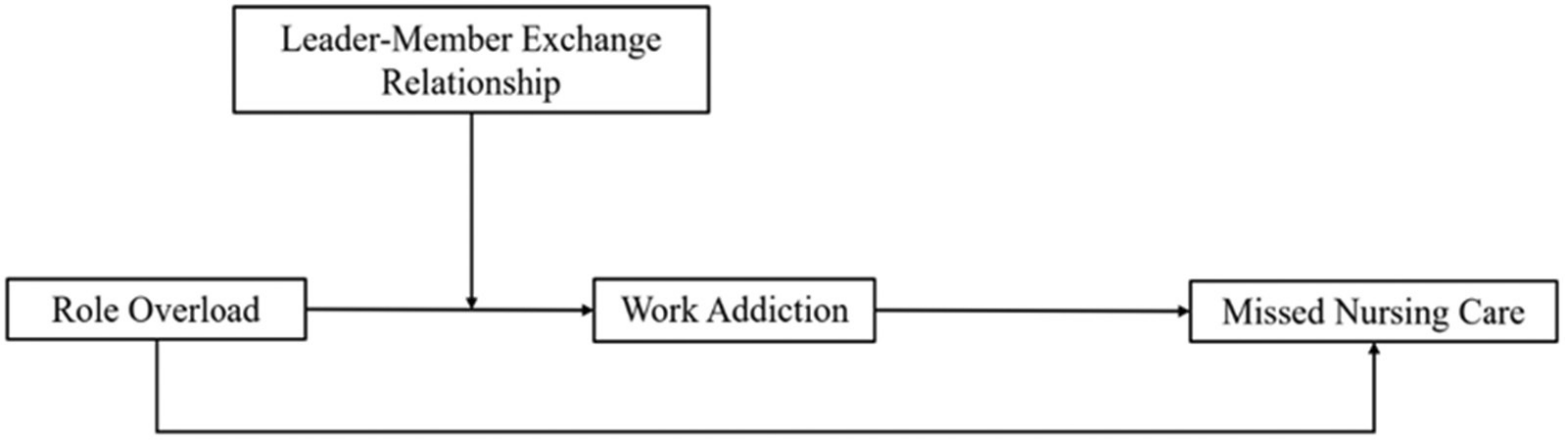
The conceptual framework.

This study aimed to complement the body of knowledge regarding factors that could buffer the negative relationship between nurse role overload and missed nursing care by examining a potential mediating variable, work addiction, and a moderator variable, leader‐member exchange. Figure 1 provides an outline of the conceptual framework for the study.

## 2 METHODS

### 2.1 Study design and data collection

We used a multisite cross‐sectional quantitative study design. Furthermore, to improve the research quality, we followed the STROBE checklist. In the first stage, five cities were randomly selected by the random number table method from five regions in the east, south, central, west and north of Sichuan province. One general tertiary hospital was chosen randomly from each city in the second stage. The eligible nurses in the study were randomly selected in three shifts (morning, evening and night).

Participants who met the inclusion criteria and agreed to participate were provided with information about the study and were free to withdraw. Inclusion criteria were as follows: registered nurses who were engaging in the clinical nursing work; nurses who had no physical or mental illness; and those with informed consent and voluntary participation in the study. Nurses were excluded if they were on leave for more than three consecutive months.

### 2.2 Measures

Data were obtained from demographic characteristics and four scales. Demographic characteristics include seven dimensions (age, gender, marital status, education, work sector, work experience and professional level). For example, the professional level is classified into three levels: primary level (Registered Nurse and Nurse Practitioner), intermediate level (supervisor nurse) and senior level (Associate Chief Nurse Practitioner and Director Nurse Practitioner).

*Role overload* Nurses' role overload was measured using the scale developed by Peterson et al. (Peterson et al., 1995), containing five‐question items. The scale has been widely validated as having good reliability by Chinese researchers (Liu et al., 2017; Wu & Peng, 2017). The five items were “My workload is too heavy,” “I really need to relieve some of my work,” “At work, I feel overloaded,” “I have taken on too many roles,” “I take on so much load that I can't guarantee the quality of my work.” The scale had a Cronbach's *α* of 0.844.

*Leader‐Member Exchange* The leader‐member exchange was measured using a scale developed by Graen et al. (1982). It contains seven questions (e.g. “In general, I know if my leader is satisfied with my performance”), and a higher score indicates a higher degree of the social exchange relationship between the subordinate and the leader (Graen et al., 1982). In this study, the scale had a Cronbach's *α* of 0.840.

*Work Addiction* The variable of work addiction was measured using the Dutch Work Addiction Scale (DUWAS), which is divided into two dimensions: Work Excessively and Work Compulsively (Schaufeli et al., 2009). It contains a total of 10 items. The scale asks subjects to report their level of agreement with item descriptions such as “At work, I seem to be in a hurry, racing against time all the time” and “It is important to me to work hard, even if I do not like the work I am doing.” The DUWAS has a Cronbach's *α* of 0.819 in this study.

*Missed nursing care* Nurses' missed nursing care was measured using the instrument *MISSCARE Survey* Part A developed by Kalisch and Williams (2009). There are 24 entries in total. The scale had a Cronbach's *α* of 0.851 in this study.

The role overload scale includes one dimension. The leader‐member exchange scale is unidimensional, and the work addiction scale includes two dimensions. Thus, data were obtained from 12 dimensions. In the statistical analysis, the sample size was about 20 times the number of dimensions, and another 20% expanded the sample to account for missing and invalid questionnaires. Therefore, the sample approximately consisted 500 nurses.

These scales have been used in previous Chinese studies. The items within the scale were randomized to avoid possible item order bias. Each item of the *MISSCARE Survey* Part A was rated on a five‐point Likert‐type scale ranging from “never missed” = 1, “rarely missed” = 2, “occasionally missed” = 3, “frequently missed” = 4, and “always missed” = 5. Each item of the rest of scales was rated on a five‐point Likert‐type scale ranging from “strongly disagree = 1, disagree = 2, neutral = 3, agree = 4, to strongly agree = 5.” All the scales mentioned above proved to have good reliability and validity in this study.

### 2.3 Data collection

Data were gathered from March 2022–May 2022. The authors contacted the nursing administrators at these hospitals before data collection. With the consent of the nursing managers, all nurse participants completed the questionnaire during working hours. The survey researcher described the study's purpose, risks and benefits, and the participants signed an informed consent form. The survey instrument was then distributed to each nurse by the researcher. Participants were informed that the purpose of this study was to learn more about nurses. Participation was voluntary, and no personally identifiable information was collected. Participants were asked to read the items carefully and answer relevant questions. Participants were informed of their right to withdraw from the survey. To ensure anonymity, participants returned completed questionnaires directly to the researcher.

Ultimately, a total of 420 questionnaires were distributed in this study. After excluding 17 invalid questionnaires (not completed at all stages of the study or with invalid answers, such as using the same answer for the entire questionnaire), 403 responses were obtained, with a valid return rate of 95.95%.

### 2.4 Data analysis

Data entry and analysis were conducted using SPSS 26.0 and Amos 24.0. First, descriptive statistics and correlation analysis were conducted on the main variables by SPSS 26.0. Second, we used Amos 24.0 to test the mediation model of work addiction in the relationship between role overload and missed nursing care by constructing a structural equation model (SEM). This study refers to De Carvalho and Chima (2014) for the fitting indicators and the criteria for the goodness of fitting indicators, as detailed in Table 2. And we also used Amos 24.0 to test the moderating mediation model through path analysis. To further show the moderating role of leadership‐member exchange between role overload and work addiction, this study referring to Hayes (2018) plotted the moderating effect figure and performed a simple slope test.

## 3 ETHICAL CONSIDERATIONS

Ethics review committee approval was obtained from the Ethics Board at Sichuan Academy of Medical Science & Sichuan Provincial People's Hospital before collecting data (No. 2022‐78). All nursing leaders and eligible nurses were informed about the study and the ethical principles that centred on human rights protection and minimal harm. Two authors answered enquires when they were made. All data were stored in a computer protected by passwords and destroyed after 5 years. No one had access to the data except the research team.

## 4 RESULTS

### 4.1 Demographic characteristics

The sample in this study included 403 nurses, most of them were women (398, 98.8%), and the average age was 32.25 ± 6.34 years. As for their educational background, most of the participants had a bachelor's degree (272, 67.5%). In total, 281 nurses are at the primary (*r* = 0.43 (Registered Nurse and Nurse Practitioner)), making up 69.7% of the sample, and their average job tenure is 10.04 ± 6.69 years.

### 4.2 Correlations between variables

Through correlation analysis, the results suggest that role overload is positively associated with missed nursing care (*r* = 0.435, *p* < 0.01); thus, Hypothesis 1 is acceptable. leader‐member exchange is negatively associated with missed nursing care (*r* = −0.480, *p* < 0.01). Moreover, work addiction is positively associated with missed nursing care (*r* = 0.632, *p* < 0.01). The results are shown in Table 1.

**TABLE 1:** Correlations between variables

	M	SD	1.	2.	3.	4.
1. Role Overload	4.086	0.655	1			
2. Leader‐Members Exchange	3.206	0.608	−0.153**	1		
3. Work Addiction	3.292	0.637	0.551**	−0.263**	1	
4. Missed Nursing Care	3.119	0.487	0.435**	−0.480**	0.632**	1

** *p* < 0.01.

### 4.3 Mediation model testing

**FIGURE 2:**
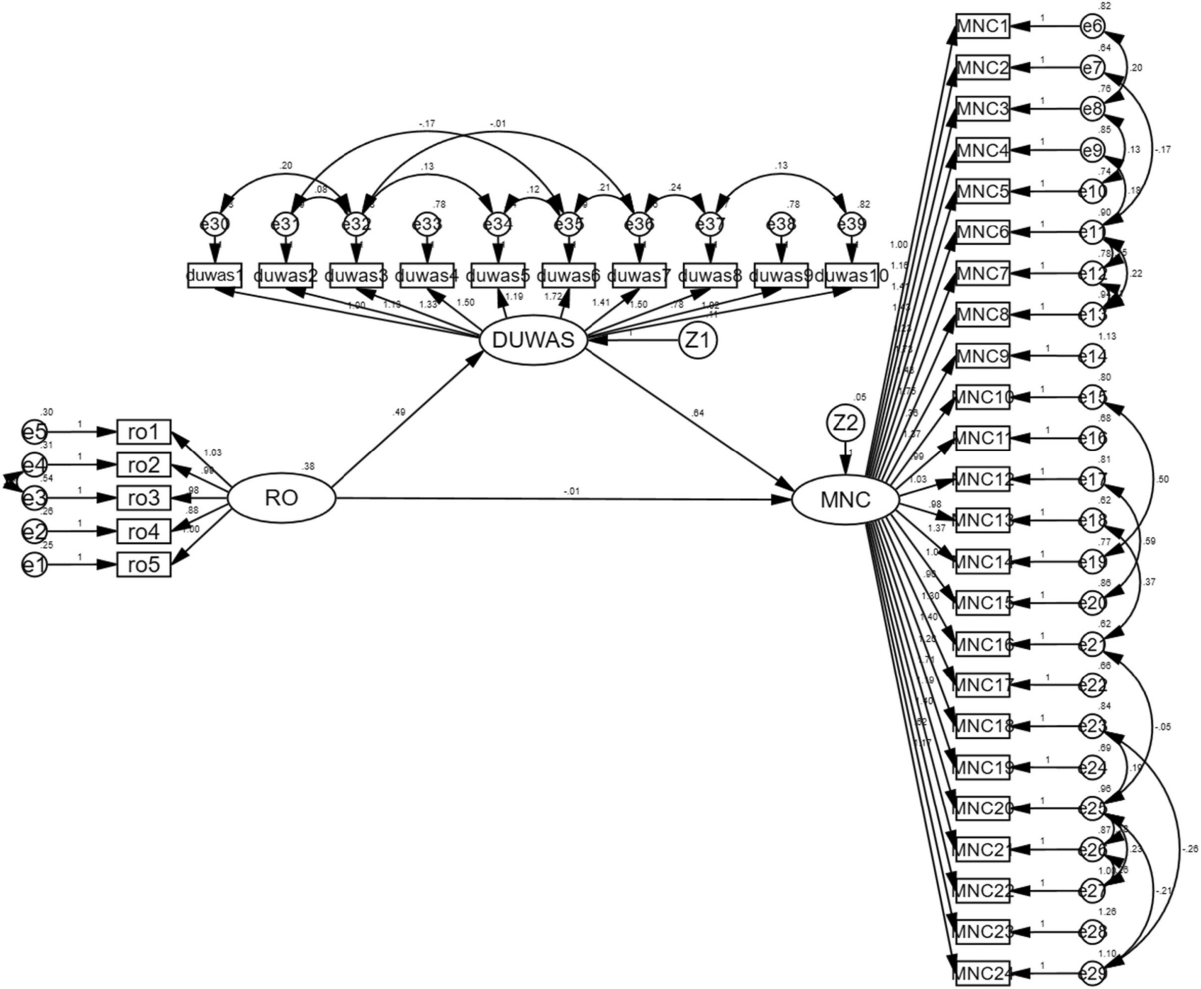
Mediation model.

This study used AMOS 26.0 to construct a mediation structural equation model with role overload as the independent variable, work addiction as the mediating variable, and missed nursing care as the dependent variable. The model diagram is shown in Figure 2. The maximum likelihood method was used to fit the structural equation model. This study refers to De Carvalho and Chima (2014) for the fitting indicators and the criteria for the goodness of fitting indicators, as detailed in Table 2. All fitting indicators of the mediation model are within the acceptable range, indicating that the model fits well.

**TABLE 2:** Mediation model fitting indicators

Test statistic	RMSEA	GFI	NFI	RFI	IFI	TLI	CFI	PNFI	PCFI
Adaptation standard	<0.08	>0.9	>0.9	>0.9	>0.9	>0.9	>0.9	>0.5	>0.5
Data efficacy result	0.058	0.944	0.940	0.913	0.931	0.911	0.929	0.671	0.752

Abbreviations: CFI, comparative fit index; GFI, goodness of fit index; IFI, incremental fit index; NFI, normed fit index; PCFI, parsimony comparative fit index; PNFI, parsimony normed fit index; RESEA, root mean square error of approximation; RFI, relative fit index; TLI, Tucker‐Lewis index.

As shown in Table 3, the results of path analysis show that role overload positively predicts work addiction (*β* = 0.681, *p* < 0.001), and work addiction positively predicts missed nursing care (*β* = 0.798, *p* < 0.001). Furthermore, the direct effect of role overload on nursing absence is not significant (*β* = −0.024, *p* = 0.756). Therefore, work addiction plays a mediation role between role overload and missed nursing care. Consequently, hypotheses 2–4 are acceptable.

**TABLE 3:** Path analysis of mediation model

Path	Estimate	SE	95% CI		*t*	*p*	*β*
			Lower	Upper			
RO → DUWAS	0.494	0.066	0.356	0.665	7.540	<0.001	0.681
DUWAS → MNC	0.643	0.126	0.400	1.020	5.086	<0.001	0.798
RO → MNC	−0.014	0.045	−0.16	0.098	−0.310	0.756	−0.024
RO → RO5	1						0.775
RO → RO4	0.880	0.062	0.754	1.018	14.305	<0.001	0.728
RO → RO3	0.980	0.081	0.819	1.154	12.057	<0.001	0.633
RO → RO2	0.986	0.069	0.839	1.163	14.230	<0.001	0.734
RO → RO1	1.035	0.069	0.899	1.197	14.890	<0.001	0.757
MNC → MNC1	1						0.368
MNC → MNC2	1.155	0.200	0.852	1.643	5.788	<0.001	0.461
MNC → MNC3	1.411	0.204	1.036	1.973	6.911	<0.001	0.503
MNC → MNC4	1.427	0.241	1.021	2.119	5.929	<0.001	0.487
MNC → MNC5	1.229	0.213	0.872	1.844	5.764	<0.001	0.456
MNC → MNC6	1.729	0.279	1.210	2.585	6.195	<0.001	0.548
MNC → MNC7	1.433	0.239	1.003	2.194	6.001	<0.001	0.503
MNC → MNC8	1.746	0.282	1.262	2.621	6.189	<0.001	0.542
MNC → MNC9	0.358	0.167	0.046	0.765	2.141	0.032	0.12
MNC → MNC10	1.367	0.231	0.974	1.999	5.907	<0.001	0.481
MNC → MNC11	0.989	0.184	0.673	1.43	5.364	<0.001	0.395
MNC → MNC12	1.034	0.197	0.694	1.559	5.259	<0.001	0.381
MNC → MNC13	0.975	0.179	0.677	1.432	5.440	<0.001	0.406
MNC → MNC14	1.369	0.23	0.962	2.023	5.948	<0.001	0.489
MNC → MNC15	1.012	0.197	0.664	1.488	5.137	<0.001	0.365
MNC → MNC16	0.958	0.177	0.649	1.452	5.397	<0.001	0.400
MNC → MNC17	1.297	0.217	0.923	1.897	5.986	<0.001	0.496
MNC → MNC18	1.398	0.238	0.986	2.082	5.885	<0.001	0.481
MNC → MNC19	1.257	0.214	0.926	1.846	5.884	<0.001	0.477
MNC → MNC20	1.706	0.279	1.226	2.550	6.123	<0.001	0.530
MNC → MNC21	1.192	0.216	0.852	1.746	5.513	<0.001	0.418
MNC → MNC22	1.399	0.245	1.082	1.958	5.711	<0.001	0.449
MNC → MNC23	0.616	0.189	0.3	1.026	3.261	0.001	0.193
MNC → MNC24	1.165	0.226	0.764	1.751	5.153	<0.001	0.371
DUWAS → duwas1	1						0.463
DUWAS → duwas2	1.134	0.154	0.878	1.524	7.383	<0.001	0.550
DUWAS → duwas3	1.328	0.148	1.070	1.742	8.983	<0.001	0.585
DUWAS → duwas4	1.500	0.192	1.130	2.059	7.802	<0.001	0.603
DUWAS → duwas5	1.187	0.16	0.895	1.644	7.407	<0.001	0.552
DUWAS → duwas6	1.723	0.215	1.316	2.426	8.026	<0.001	0.653
DUWAS → duwas7	1.413	0.181	1.074	1.998	7.823	<0.001	0.614
DUWAS → duwas8	1.501	0.196	1.157	2.080	7.664	<0.001	0.584
DUWAS → duwas9	0.783	0.136	0.540	1.157	5.768	<0.001	0.368
DUWAS → duwas10	1.023	0.155	0.766	1.372	6.605	<0.001	0.451

Abbreviations: CI, confidence interval; SE, standard error.

### 4.4 Moderating effects testing

**FIGURE 3:**
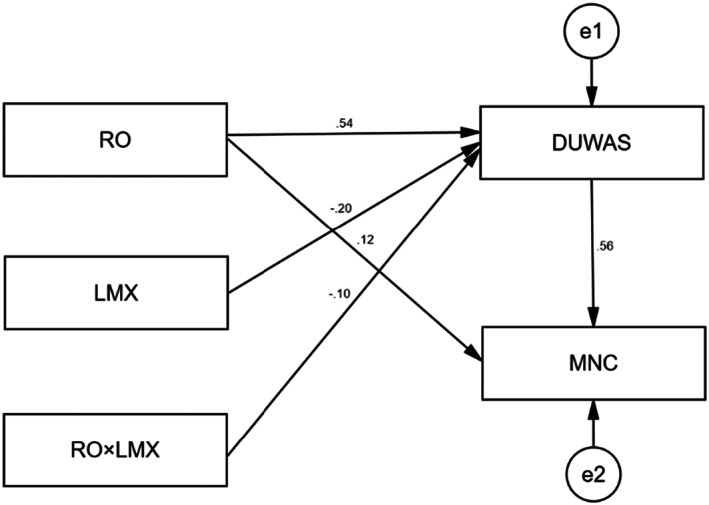
Moderated mediation model.

This study used AMOS 26.0 to construct a moderated mediation model. And the interaction term is the value of the multiplication of the standardized mean of role overload and the standardized mean of leadership‐member exchange. The model diagram is shown in Figure 3. This study refers to De Carvalho and Chima (2014) for the fitting indicators and the criteria for the goodness of fitting indicators, as detailed in Table 4. All fitting indicators of the mediation model are within the acceptable range, indicating that the model fits well.

**TABLE 4:** Moderated mediation model fitting indicators

Test statistic	RMSEA	GFI	NFI	RFI	IFI	TLI	CFI	PNFI	PCFI
Adaptation standard	<0.08	>0.9	>0.9	>0.9	>0.9	>0.9	>0.9	>0.5	>0.5
Data efficacy result	0.097	0.991	0.982	0.911	0.986	0.928	0.986	0.796	0.797

Abbreviations: CFI, comparative fit index; GFI, goodness of fit index; IFI, incremental fit index; NFI, normed fit index; PCFI, parsimony comparative fit index; PNFI, parsimony normed fit index; RESEA, root mean square error of approximation; RFI, relative fit index; TLI, Tucker‐Lewis index.

**FIGURE 4:**
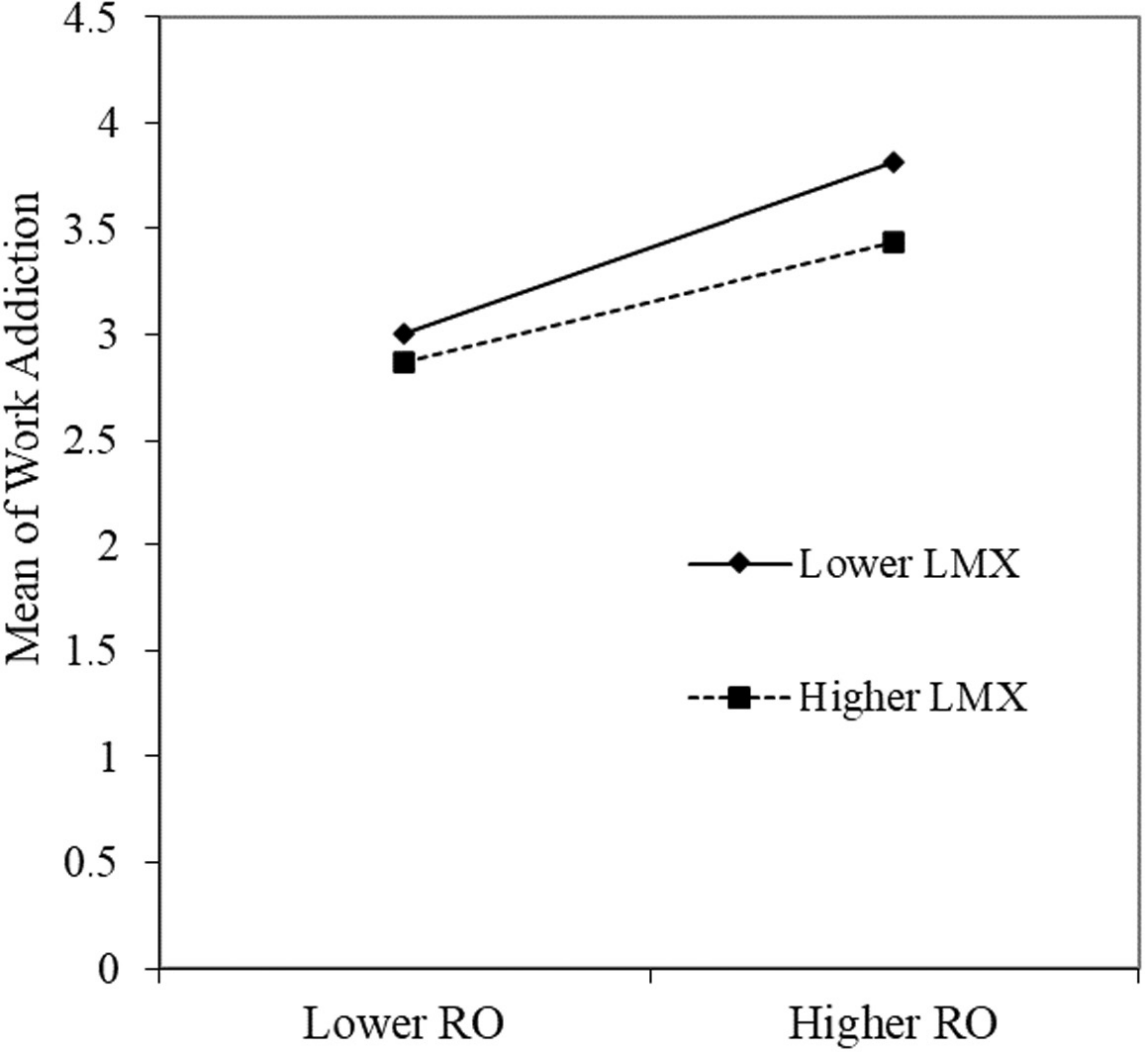
Moderating effect.

As shown in Table 5, the results of path analysis show that leader‐member exchange negatively predicts work addiction (*β* = −0.200, *p* < 0.001), and the interaction term between leader‐member exchange and role overload negatively predicts work addiction (*β* = −0.102, *p* < 0.05). It suggests that leader‐member exchange plays a moderating role between role overload and work addiction. To further show the moderating role of leadership‐member exchange between role overload and work addiction, this study referring to Hayes (2018) categorized leadership‐member exchange and role overload as higher group (M + 1 SD) and lower group (M − 1 SD) and plotted the moderating effect figure (see Figure 4). The simple slope test shows that the effect of role overload on work addiction is stronger when the level of leader‐member exchange is lower (*b* = 0.407, *t* = 11.061, *p* < 0.001), and it is weaker when the level of leader‐member exchange is higher (*b* = 0.285, *t* = 7.745, *p* < 0.001). Therefore, Hypothesis 5 is acceptable.

**TABLE 5:** Path analysis of moderated mediation model

Path	Estimate	SE	95% CI		*t*	*p*	*β*
			Lower	Upper			
LMX → DUWAS	−0.209	0.042	−0.283	−0.122	−4.957	<0.001	−0.200
RO → DUWAS	0.528	0.039	0.418	0.633	13.472	<0.001	0.544
RO × LMX → DUWAS	−0.061	0.024	−0.106	−0.016	−2.516	0.012	−0.102
DUWAS → MNC	0.431	0.035	0.339	0.528	12.354	<0.001	0.564
RO → MNC	0.092	0.034	0.013	0.179	2.722	0.006	0.124

Abbreviations: CI, confidence interval; SE, standard error.

## 5 DISCUSSION

This study aimed to investigate the relationship between aspects of role overload, work addiction, leader‐member exchange and missed nursing care. Our findings suggested that work addiction mediated the relationship between role overload and missed nursing care. In addition, we found that leader‐member exchange moderated the relationship between role overload and missed nursing care. Higher quality leader‐member exchange buffers the negative effects of role overload on aspects of missed nursing care. These findings provide important insights for researchers and nursing managers. We expand on these findings' theoretical and practical implications and offer perspectives for future research.

Initially, our results confirmed Hypothesis 1, which stated that role overload exacerbated missed nursing care. These findings are consistent with the earlier study (Rosenberg, 2019). This is probably the result of an imbalance where the demands of the role far outweigh the resources of the individual, thus making nurses face more obstacles to meet the needs of patients.

Secondly, Hypothesis 2 suggested that role overload would accelerate work addiction. The results of this study are consistent with our conceptual framework and the majority of empirical findings (Falco et al., 2020; Spagnoli et al., 2020). Job demands positively predict work addiction, when employees face overload in their role, they may generate negative affectivity, perceive a suboptimal work environment, and develop negative motivation to work, thus leading to overwork (Malinowska et al., 2018; Molino et al., 2016; Van Wijhe et al., 2011).

Notably, our results confirmed that work addiction is positively correlated with missed nursing care. The possible reason is that work addiction can affect work performance through excessive individual attrition and physical and psychological costs. At the individual level, work addiction often indirectly affects job performance through workload and emotional exhaustion (Xu et al., 2021). In addition, work addiction among nurses can indirectly lead to lower job performance due to work–family conflict (Gillet et al., 2021). In a survey of 1781 nurses (Andreassen et al., 2018) found that work addiction was significantly and positively associated with negative work‐related events. These findings are supportive of the fact that work addiction compounds missed nursing care.

The results supported Hypothesis 4, which suggested that role overload was indirectly related to missed nursing care through work addiction, and work addiction plays a completely mediating role between role overload and missed nursing care. There was no evidence of a direct effect of role overload on missed nursing care. Such results suggest that role overload influences missed nursing care primarily through work addiction. This may be because that shortages of organizational (e.g. social support) or individual (e.g. self‐efficacy) resources may be compensated for by overwork. When resources are not effectively replenished, it is difficult for individuals to recover physically and mentally, leading to a downward cycle of resource depletion (Hobfoll, 2001). When role demands are higher than nurses' expectations, they tend to mitigate the loss of resources by overworking to meet job demands as much as possible to the point of going out of control. As a result of negative work responses, nurses become chronically addicted to work (Clark et al., 2020), leading to their inability to recover physically and mentally, further exacerbating the loss of resources and eventually resulting in missed nursing care.

H5 leader‐member exchange plays a moderating role between role overload and work addiction. Specifically, the effect of role overload on work addiction is weaker when the level of leader‐member exchange is higher. The findings confirmed this hypothesis. Similar to the previous study (Afota et al., 2021), role overload on work addiction is likely greater for nurses with low‐quality leader‐member exchange than those with a high one. From the standpoint of COR theory (Hobfoll, 2001), high‐quality leader‐member exchange can nourish the resource base of employees, which suggests that leader‐member exchange may enable them to handle their workload better.

Our results from structural equation modelling suggest that leader‐member exchange is a vital work resource in the workplace that mitigates the potential threat of role overload on missed nursing care. In other words, nurses with high leader‐member exchange levels are expected to have more resources at work to facilitate the achievement of their desired work goals, resulting in improved performance. This is consistent with the findings of Martin et al, who found a positive correlation between leader‐member exchange and task performance (Martin et al., 2016). The perception of high‐quality leader‐member exchange makes nurses more identified with their organization and more aligned with the nursing work they are currently engaged in, thus providing more conditions for reducing missed nursing care. These results are consistent with the relevant study (Miao et al., 2020). Our findings suggest that positive hierarchical relationships can protect staff resources through social exchange and prevent the depleting influence of work stressors on staff. When in a resource‐constrained environment, employees' perception of leader‐member exchange was sensitive. Also, the negative impact of work addiction on job performance through emotional exhaustion was probably stronger when leadership recognition of nurses' work was low (Sandrin et al., 2019). However, nurses can be motivated to provide nursing care despite high workloads, which is similar to the existing study (Srulovici & Yanovich, 2022). This study highlights the significance of exploring the leader‐member‐exchange relationship as a boundary condition that enables employees to work effectively in a stressful work environment and reduces the negative effects of role overload on nurses, thereby reducing missed nursing care.

These findings have practical implications for promoting organizational support in Chinese hospitals. This study contributes to the literature in several ways. First, although existing nursing research has identified some factors that influence missed nursing care, little academic attention has been paid to role stressors, particularly role overload. This study suggests that nurses view role overload more as a hindering job demand than a challenging job demand because it seriously threatens nurses' ability to perform effectively. Such perceptions ultimately exacerbate work addition and missed nursing care. Secondly, these findings expand the scope of research on the aftereffects of role overload and provide a basis for in‐depth research on the factors influencing missed nursing care. Third, compared to previous nursing research, the most significant contribution of this study is that it provides explicit answers to the confusion of whether and how role overload is related to missed nursing care, especially by highlighting the potential role of leader‐member exchange as a buffer. Leader‐member exchange can increase nurses' intrinsic motivation to help the organization achieve its goals and increase emotional commitment to the organization (Srulovici &Yanovich, 2022). When nurses perceive higher levels of leader‐member exchange, they may become more engaged in their work to improve their task performance, thereby reducing the draining effects of role overload.

## 6 CONCLUSION

Based on the JD‐R model, this study examined a critical process, work addiction, which explains how role overload exacerbates missed nursing care and examined the buffering role of leader‐member exchange. The findings suggest that work addiction plays a crucial role in the relationship between role overload and missed nursing care. Furthermore, leader‐member exchange acts as an environmental resource that mitigates the effects of role overload on missed nursing care.

## 7 LIMITATIONS AND FUTURE RESEARCH

Several limitations may affect the plausibility of the study results. First, this study was impossible to obtain causality explanations because this was a cross‐sectional study. Second, we used a self‐report measure of the sample. Although self‐reporting is the most appropriate method to assess nurses' perceptions, it may have somehow led to an artificial exaggeration of the relationships found in this study leading to response bias in this research. Finally, we found that leader‐member exchange is an essential resource in the workplace and that advanced variables such as multilevel modelling will be needed in the future to confirm the results of this study.

## 8 IMPLICATIONS FOR NURSING MANAGEMENT

The results obtained in this study have several functional, practical implications for nursing managers. First, practitioners should understand that role overload may exacerbate missed nursing care. Therefore, nursing managers can minimize the influence of factors that contribute to role overload. In this context, setting reminders of the most critical priorities may help reduce role overload, and training on dealing with overload is crucial.

In addition, these results regarding the moderating role of leader‐member exchange in the relationship between role overload and missed nursing care have some practical implications. First, in a resource‐stressed environment, nursing managers should continuously improve their leadership and acquire the skills to interact well with their subordinates, thus enabling nurses to obtain high‐quality leader‐member exchange, especially for young and junior nurses. Secondly, encouraging nurses to build harmonious interpersonal relationships is an efficient way to help them get high‐quality leader‐member exchange. Finally, managers need to focus on organizing activities for their members to increase workplace fun and foster friendships among nurses to increase team cohesion and maintain team performance.

## AUTHOR CONTRIBUTIONS

Design the experimented and received research funding: Linli Xie, Wenchun Jiang, Jie Jing, and Eksiri Niyomsilp. Data collection: Linli Xie, Wenchun Jiang, Lu Feng, Yilin Wen, Li Wang, and Rongmei Zheng. Data analysis and interpretation: Linli Xie, Wenchun Jiang, and Jie Jing. Drafting of the article: Linli Xie, and Wenchun Jiang. Critical revision of the article: Linli Xie, Wenchun Jiang, Jie Jing, and Eksiri Niyomsilp.

## FUNDING INFORMATION

This study was funded by the Health Commission of Sichuan Province, China (Grant number: 21PJ075).

## CONFLICT OF INTEREST

The author(s) declared no potential conflicts of interest with respect to the research, authorship, and/or publication of this article.

## ETHICAL CONSIDERATIONS

Ethical conformity approval was obtained from the Ethics Board at Sichuan Academy of Medical Science & Sichuan Provincial People's Hospital (No.2022–78, issued on February 22, 2022).

## Data Availability

The datasets generated and/or analyzed during the current study are not publicly available due to privacy guidelines but are available from the corresponding author on reasonable request.
